# Effects of meiotic stage-specific oocyte vitrification on mouse oocyte quality and developmental competence

**DOI:** 10.3389/fendo.2023.1200051

**Published:** 2023-06-26

**Authors:** Dongmei Deng, Juan Xie, Yin Tian, Ling Zhu, Xuemei Liu, Junxia Liu, Guoning Huang, Jingyu Li

**Affiliations:** ^1^ Chongqing Key Laboratory of Human Embryo Engineering, Center for Reproductive Medicine, Chongqing Health Center for Women and Children, Chongqing, China; ^2^ Chongqing Clinical Research Center for Reproductive Medicine, Center for Reproductive Medicine, Women and Children’s Hospital of Chongqing Medical University, Chongqing, China

**Keywords:** germinal vesicle oocyte, oocyte vitrification, fertility preservation, *In vitro* maturation, developmental competence

## Abstract

**Introduction:**

Acquisition of germinal vesicle (GV) stage oocytes for fertility preservation (FP) offers several benefits over in vivo matured oocyte cryopreservation following ovarian stimulation, particularly for cancer patients necessitating immediate treatment. Two FP approaches for GV oocytes are available: vitrification before in vitro maturation (IVM) at the GV stage (GV-VI) or post-IVM at the metaphase II (MII) stage (MII-VI). The optimal method remains to be determined.

**Methods:**

In this study, mouse oocytes were collected without hormonal stimulation and vitrified either at the GV stage or the MII stage following IVM; non-vitrified in vitro matured MII oocytes served as the control (CON). The oocyte quality and developmental competence were assessed to obtain a better method for immediate FP.

**Results:**

No significant differences in IVM and survival rates were observed among the three groups. Nevertheless, GV-VI oocytes exhibited inferior quality, including abnormal spindle arrangement, mitochondrial dysfunction, and early apoptosis, compared to MII-VI and CON oocytes. Oocyte vitrification at the GV stage impacted maternal mRNA degradation during IVM. In addition, the GV-VI group demonstrated significantly lower embryonic developmental competence relative to the MII-VI group. RNA sequencing of 2-cell stage embryos revealed abnormal minor zygotic genome activation in the GV-VI group.

**Conclusion:**

Vitrification at the GV stage compromised oocyte quality and reduced developmental competence. Consequently, compared to the GV stage, oocyte vitrification at the MII stage after IVM is more suitable for patients who require immediate FP.

## 1 Introduction

Fertility preservation (FP) has become a standard approach for cryopreserving gametes or embryos for future reproductive purposes, particularly for women at risk of premature ovarian insufficiency due to cancer treatments, autoimmune or hematological diseases (1–4). Assisted reproductive technology (ART) has made FP possible, and oocyte vitrification has emerged as an effective method for both married and unmarried women (5, 6). However, FP in cancer patients remains a contentious issue due to the potential delay of anti-tumor therapy caused by controlled ovarian stimulation (COS) (1). Moreover, hormones administered during COS could stimulate hormone-sensitive malignant cell proliferation, leading to a worsened prognosis (2). In these cases, retrieval of oocytes at the GV stage without COS, followed by a combination of *in vitro* maturation (IVM) and vitrification, can achieve FP without delaying cancer treatment and circumvent potential hormone treatment adverse effects (7).

Currently, two primary methods are employed for FP with IVM: oocyte vitrification at the germinal vesicle (GV) or metaphase II (MII) stages following IVM. Numerous studies have explored the efficacy of vitrification at varying meiotic stages. Several studies analyzing GV oocytes collected from stimulated cycles revealed that oocytes vitrified at the MII stage post-IVM exhibited enhanced potential for maturation and embryonic development compared to oocytes vitrified at the GV stage (8, 9). Conversely, Molina et al. observed higher IVM rates and superior embryonic development when oocytes were vitrified at the GV rather than MII stage (10). In addition, a recent meta-analysis indicated that the timing of vitrification, either before or after IVM, did not influence the survival, maturation, fertilization, or development rates of oocytes obtained from stimulated cycles (11). Thus, clinical data on stimulated cycles remains equivocal. Greater attention should be placed on GV oocytes retrieved without COS, as most cancer patients rely on unstimulated cycles for FP. To date, only two studies have compared embryological outcomes between oocytes vitrified from unstimulated cycles at the GV and MII stages following IVM, with similar IVM rates and blastocyst formation observed across groups (12, 13). However, a bovine study showed that vitrifying GV oocytes from unstimulated ovaries resulted in better blastocyst formation than vitrifying at the MII stage post-IVM (14). Consequently, additional research utilizing GV oocytes retrieved without hormonal stimulation is required to determine the optimal IVM FP approach.

In this study, we obtained GV oocytes from unstimulated mice and performed vitrification at either the GV or MII stages following IVM. We assessed the quality of oocytes vitrified at distinct stages based on criteria such as mitochondrial function, spindle morphology, and maternal mRNA degradation. Furthermore, we employed RNA sequencing (RNA-Seq) to investigate the mechanisms driving the variations in developmental competence. Based on the above results of research utilizing GV oocytes retrieved without hormonal stimulation, an optimal IVM FP approach can be determined.

## 2 Materials and methods

### 2.1 Animals and ethics approval

This study utilized female ICR mice, Six-week-old, which were obtained from SPF Biotechnology Company (Beijing, China). The mice were housed and maintained under standard conditions (20-22°C, 50–70% humidity, 12/12-h light/dark cycle) with the food and water provided ad libitum throughout the study period. All animal care and experimental procedures were conducted in accordance with the Animal Research Committee guidelines of the Chongqing Health Center for Women and Children (The ethics committee approval number: 2022001).

### 2.2 Oocyte collection

Following a 2-week adaptation period, female ICR mice were sacrificed and their ovaries were carefully dissected. The ovaries were gently minced using fine blades, and naked GV oocytes were released from the follicles into prewarmed M2 medium (Sigma, St.Louis, MO, USA).

### 2.3 *In vitro* maturation

The *in vitro* maturation protocols used in this study were based on previously described methods by *Zhang et al.* (15). In brief, the naked GV oocytes were cultured in G1 medium (Vitrolife, Gothenburg, Sweden) for 16 hours at 37 °C in humidified air environment containing 6% CO2. The maturation of oocytes was assessed by monitoring the germinal vesicle breakdown (GVBD) and extrusion of the first polar body (PB1).

### 2.4 Vitrification-warming procedure

Oocyte vitrification was conducted at room temperature using a commercial kit (Kitazato, Shizuoka, Japan). First, oocytes were transferred to a 60-µL mixture of equilibration solution (ES) and washing solution 2 (WS2) at a ratio of 1:1 for 3 min. Second, 30 µL ES was added to the mixture described in the first step and the oocytes were incubated in the new mixture for 3 min. Third, the oocytes were removed and placed in ES for 9 min. Then, they were exposed to vitrification solution (VS) for 45–60s. Finally, the oocytes were loaded onto the tip of a Cryotop carrier with a minimal volume of VS and immediately immersed in liquid nitrogen. The oocytes were stored in liquid nitrogen for at least 1 week.

Oocyte warming was performed using a four-step procedure. In step 1, the vitrified oocytes on the tip of the Cryotop carrier were dipped into a thawing solution (TS) that had been preheated to 37 °C for 1 h, and held for 1 min. In step 2, the oocytes were suspended in a diluent solution (DS) for 3 min. In steps 3 and 4, the oocytes were removed and placed into WS1 and WS2, respectively, for 5 min each. Finally, the oocytes were transferred to G1 medium for IVM after washing three times with the G1 medium. The oocytes were considered to have survived if they showed no signs of degeneration after warming for 2 h. Degeneration was characterized by darkened or retracted ooplasm.

### 2.5 Intracytoplasmic sperm injection

The ICSI procedure was described previously by Stein, *P et al.* (16). Briefly, fresh sperm collected from the cauda epididymis were cultured for 15 min in a 100 µL drop of M2 medium with cytochalasin-B (1:200; Sigma) covered with mineral oil (Vitrolife). Individual sperm heads were separated through the application of a few piezo pulses and then injected into MII oocytes after IVM or vitrification-warming with a piezo micromanipulator. After ICSI, surviving MII oocytes were cultured in a KSOM medium (Sigma). Oocytes with two pronuclei and a second polar body were considered fertilized eggs.

### 2.6 Embryo culture and time-lapse monitoring

The EmbryoScope (Vitrolife) was used for embryo culture. Briefly, once injected, the oocytes were transferred to pre-equilibrated KSOM medium in 25-µL micro drops, overlaid with 1.5 mL mineral oil and incubated at 37 °C under 6% CO2, 5% O2, and 89% N2.

Images of each embryo were analyzed using the EmbryoViewer image analysis software. Embryonic developmental events were recorded in terms of hours since ICSI, including the time to fading of the two-pronuclei (t2PNf), times to a 2-cell, 4-cell, and 8-cell embryos (t2, t4, and t8, respectively); time to the formation of the morula (tm), and time to the formation of the blastocyst (tb).

### 2.7 Assessment of mitochondrial function

Assessment of mitochondrial function was conducted through fluorescent staining of live MII oocytes. The mitochondrial membrane potential (Δφm) of oocytes was measured through JC-1 staining (Beyotime, Shanghai, China, C2006). The examined oocytes in each group were exposed to JC-1 (1:500) in M2 medium at 37°C for 20 min. The level of mitochondrial reactive oxygen species (ROS) in oocytes was measured using the MitoSOX™ Red (ThermoFisher Scientific, Waltham, MA, USA, M36008). The oocytes examined in each group were stained with 5 μM MitoSOX at 37°C for 20 min. The level of ROS generated in oocytes was measured using the oxidation-sensitive fluorescent probe 2′,7′-dichlorofluorescein diacetate (DCFH-DA; Beyotime, #S0063). The oocytes examined were stained with DCFH-DA (1:500) at 37°C for 30 min. Calcium levels in oocytes were measured using the Ca^2+^-sensitive fluorescent probe Fluo-4 AM (Beyotime, S1060). The oocytes examined in each group were stained with 5 μM Fluo-4 AM at 37°C for 30 min.

The oocytes were placed on a glass-bottomed dish after three washes in fresh M2 medium. The fluorescence intensity of the oocyte was measured using laser scanning microscopy (TCS SP8; Leica, Wetzlar, Germany). The experiments were repeated three times independently. The resulting photographs were analyzed using ImageJ software (NIH, Bethesda, MD, USA).

### 2.8 Assessment of early apoptosis

Live MII stage oocytes from the three groups (CON, GV-VI, MII-VI) were incubated with annexin-V-fluorescent isothiocyanate (FITC; 1:40) (ThermoFisher Scientific, 331200) in binding buffer for 25 min at room temperature. After washing three times with fresh M2 medium, the fluorescent signals were examined with a confocal laser scanning microscope (TCS SP8; Lecia). The green fluorescence signal of annexin-V in the membrane and zona pellucida (ZP) indicates early apoptosis of oocytes, whereas fluorescence only in the ZP indicates normal oocytes. The percentage of oocytes with early apoptosis was determined. The experiments were repeated three times independently.

### 2.9 Assessment of the distribution of mitochondria

Mitochondria were stained using Mitochondria Tracker Red (Beyotime, C1049B). The staining protocol was followed as per the manufacturer’s instructions, and a concentration of 25 nM was used for staining. Live oocytes were incubated with the stain for 15 min in an incubator (37 °C and 6.0% CO2), in M2 medium containing Mito-tracker. After staining, the oocytes were washed three times in a fresh M2 medium. Finally, the oocytes were placed in a glass-bottomed dish and observed under a confocal laser scanning microscope (TCS SP8; Leica).

### 2.10 Immunofluorescence microscopy

After removal of the ZP with 0.5% HCl, oocytes were fixed with 4% paraformaldehyde (PFA) for 30 minutes and then permeabilized for 15 min with 0.3% Triton X-100 in phosphate-buffered saline (PBS). After blocking PBS-polyvinyl alcohol (PVA) solution supplemented with 3% bovine serum albumin (BSA) for 1 hour at room temperature, oocytes were incubated overnight at 4 °C with 1:500 anti-α-tubulin- FITC antibody (Sigma, F2168) for assessment of spindle morphology, as well as with 1:100 5′-FITC-oligonucleotide(dT)20 probe (Cell Signalling Technology, Beverly, MA, USA) for 1 hour at 42 °C for assessment of polyadenylated mRNA. In the final incubation step, DNA was stained for 15 min with Hoechst 33342 (1:500; Beyotime, C1022). Finally, oocytes were mounted on glass slides and viewed under a confocal laser scanning microscope (TCS SP8; Lecia). The experiments were repeated three times independently. The photographs were analyzed using ImageJ software (NIH).

### 2.11 RNA-Seq

The procedure used for RNA-Seq was described in our previous study (17). Briefly, 15 2-cell embryos from each group were prepared for RNA-Seq analysis. The Smart-Seq2 method was used for amplification, and the Qubit^®^ 3.0 Fluorometer (ThermoFisher Scientific) and 2100 Bioanalyzer (Agilent, Santa Clara, CA, USA) were used to determine the quality of the cDNA product, and ensure that its length was approximately 1–2 kb. The library was prepared following the manufacturer’s instructions (Cat. FC-131–1024; Illumina, San Diego, CA, USA) and checked with the LabChip^®^ GX Touch and Step OnePlus™ Real-Time PCR System (PerkinElmer, Waltham, MA, USA). Finally, libraries were sequenced on the Illumina HiSeq 4000 platform with 150-bp paired-end reads. Three parallel experiments were conducted for each group.

### 2.12 RNA-Seq data processing

Fastp was used to remove raw sequence reads containing adapters and poor-quality reads. HISAT2 (version 2.1.0) was used to map clean reads to the mouse reference genome (mm10). FeatureCounts (version 2.0.1) was used to obtain the read count of genes based on the annotation file, which was downloaded from the Ensembl database. Differential expression analysis was performed using the DESeq2 (version 1.30.0) R package, and genes with significant changes (absolute log10 fold change ≥ 1 and q-value < 0.05) were considered differentially expressed genes (DEGs).

We performed Gene Ontology (GO) analysis and Kyoto Encyclopedia of Genes and Genomes (KEGG) pathway analysis using the DAVID web tool with the default parameter settings. Lists of minor and major zygotic genome activation (ZGA) genes were obtained from the study of *Park SJ et al.* (18).

### 2.13 Detection of protein synthesis

As described by *Fan et al.* (19), MII oocytes were incubated in G1 medium with 100 mM L-homopropargylglycine (HPG; a methionine analog incorporated into nascent proteins during active protein synthesis) for 1 h after removal of the ZP and then fixed for 30 min at room temperature in 4% PFA. HPG signals were assessed using the Click-iT^®^ HPG Alexa Fluor^®^ Protein Synthesis Assay Kit (ThermoFisher Scientific, C10428). The mean intensity of the HPG signal was measured across the middle of each oocyte with a confocal laser scanning microscope (TCS SP8; Leica) and quantified using ImageJ software (NIH).

### 2.14 Quantitative reverse transcription polymerase chain reaction

RNA was extracted from oocytes using the ArotursTM PicopureTM RNA Isolation Kit (ThermoFisher Scientific), following the manufacturer’s instructions. Then, reverse transcription was performed using PrimeScript RT Master Mix (Takara, Dalian, China) and Power SYBR Green PCR Master Mix (Life Technologies), with the CFX-96 Touch Real-Time PCR system and the following primers:

Ybx1-F GGTCCTCCACGCAATTACCA
Ybx1-R CCTTCGGAATCGTGGTCTGT
Zar1-F GTTCTGCCGAGTGTGTGAGA
Zar1-R CAGGCGTTTGTCCTTGCATC

### 2.15 Statistical analysis

For each experiment, at least three replicates were conducted. The quantized data were expressed as mean ± standard error of the mean (SEM) and analyzed by one-way ANOVA with Games-Howell’s multiple comparisons test with GraphPad Prism 8.02 (GraphPad Software Inc., San Diego, CA, USA). Rates and frequencies are expressed as the mean value and were analyzed using the χ2 test in SPSS 25.0 (IBM Corp., Armonk, NY, USA). P values < 0.05 were considered statistically significant.

## 3 Results

### 3.1 Vitrification at GV or MII stages does not affect IVM and survival rates

**Figure 1:**
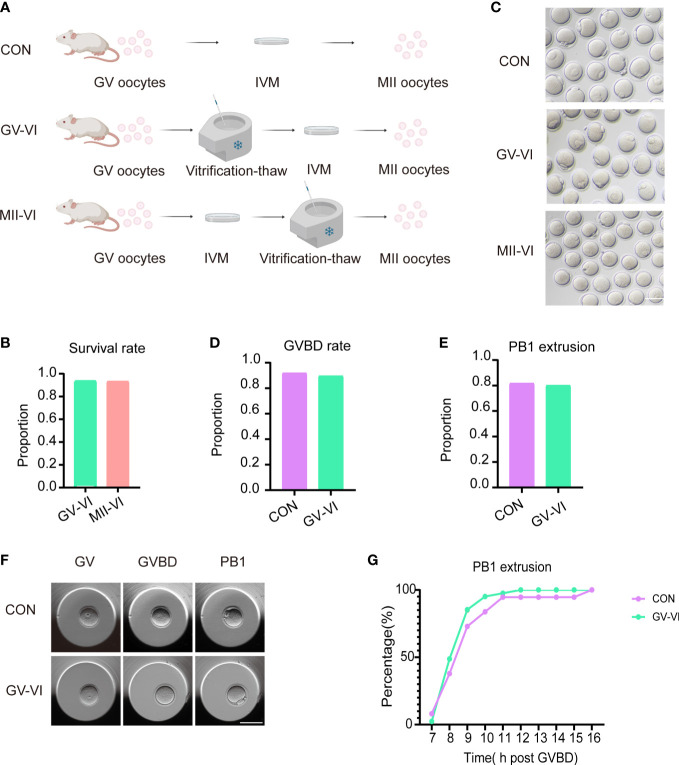
Vitrification at distinct meiotic stages did not affect IVM or post-warming survival. **(A)** Schematic representation of vitrification-warming procedures for oocytes at varying meiotic stages. CON: IVM only; GV-VI: vitrification at the GV stage before IVM; MII-VI: vitrification at the MII stage following IVM. **(B)** Survival rates were assessed in the GV-VI (n = 114) and MII-VI (n = 106) groups. **(C)** Representative images of MII- stage oocytes from the CON, GV-VI, and MII-VI groups. Scale bar, 80 μm. **(D)** and **(E)** GVBD and PB1 extrusion rates for the CON (n = 137), GV-VI (n = 146) groups. **(F)** Representative images of oocytes at various meiotic stages during IVM, captured *via* time-lapse monitoring. Scale bar, 80 μm. **(G)** The proportion of PB1 extrusion in the three groups at distinct time points post-GVBD. No significant differences were observed in the data presented in **(C–E, G)**, as determined by the χ2 test.

GV oocytes were randomly allocated into three groups according to the meiotic stage of the vitrified oocytes: CON (IVM only), GV-VI (oocyte vitrification at the GV stage before IVM), and MII-VI (oocyte vitrification at the MII stage following IVM) (**Figure 1A**). No significant difference in survival rate between GV-VI and MII-VI groups were observed (93.9% vs. 93.4%, p = 0.888; **Figure 1B**). The impact of vitrification on IVM potential was then assessed, revealing similar levels of GVBD (92.0% vs. 89.7%, p = 0.779) and PB1 extrusion (81.8% vs. 80.1%, p = 0.924) between the CON and GV-VI groups (**Figures 1C–E**). Time-lapse monitoring suggested that vitrification at the GV stage might slow maturation, although this finding was not statistically significant (**Figures 1F, G**). Overall, the results indicated that vitrification at different meiotic stages did not affect the survival rates or the IVM process.

### 3.2 Vitrification at GV stage influences mitochondrial ROS, early apoptosis, and spindle morphology

**Figure 2:**
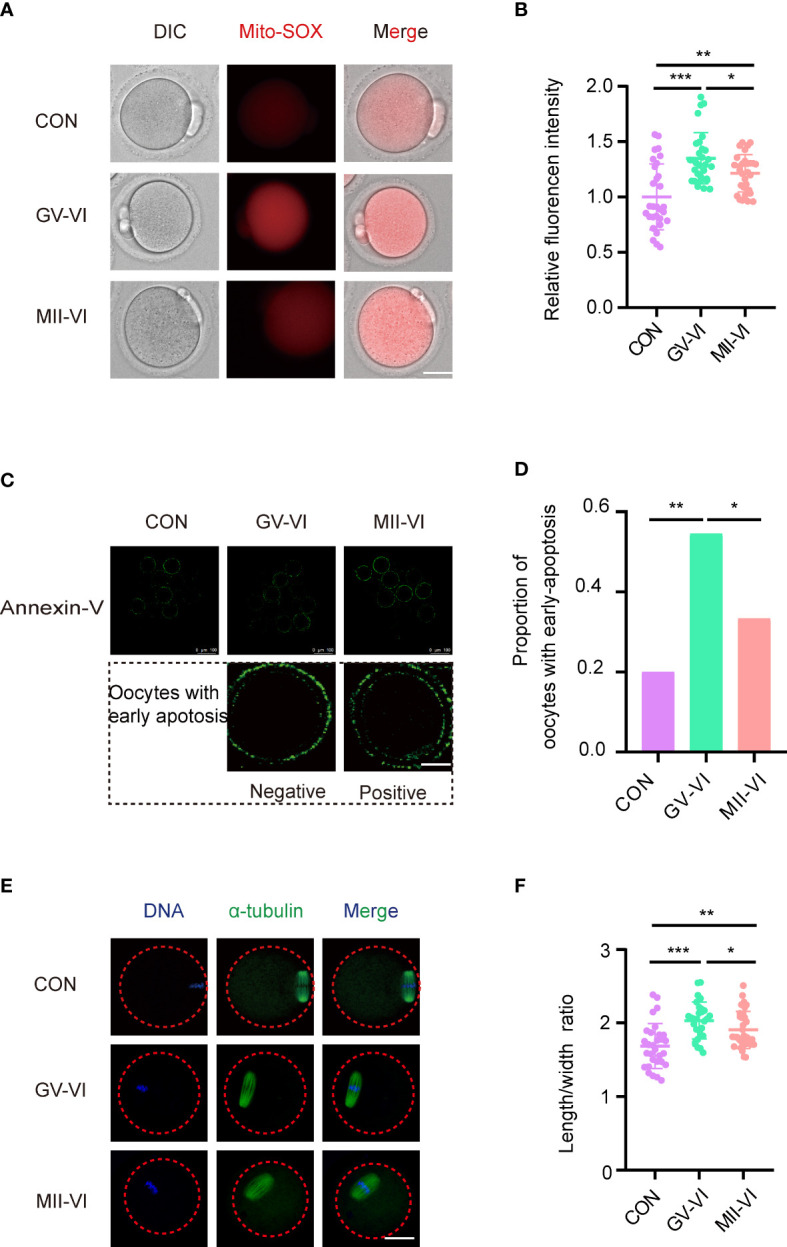
Effects of vitrification at various meiotic stages on mitochondrial ROS, early apoptosis, and spindle morphology. **(A)** Representative images of mitochondrial ROS levels were detected through MitoSOX staining of oocytes from the CON, GV-VI, and MII-VI groups. Scale bar, 25 μm. **(B)** Fluorescence intensity of mitochondrial ROS signals in oocytes of the CON (n = 30), GV-VI (n = 30), and MII-VI (n = 30) groups. **(C)** Representative images showing the apoptotic status, assessed by annexin-V staining, of oocytes in the CON, GV-VI, and MII-VI groups. Scale bar, 25 μm. **(D)** Percentage of oocytes with early apoptosis in the CON (n = 45), GV-VI (n = 44), and MII-VI (n = 42) groups. **(E)** Representative images of spindle assembly and chromosome alignment of oocytes in the CON, GV-VI, and MII-VI groups. Scale bar, 25 μm. **(F)** The ratio of spindle length to spindle width of oocytes in the CON (n = 30), GV-VI (n = 29), and MII-VI (n = 33) groups. The data in **(B, F)** are presented as the mean ± SEM, from at least three independent experiments. *p < 0.05, **p < 0.01, ***p < 0.001.

To determine the effect of vitrification at distinct meiotic stages on oocyte quality, we assessed ROS, early apoptosis, and spindle morphology at the MII stage. Both vitrification groups exhibited increased mitochondrial ROS levels, with GV-VI oocytes displaying significantly higher levels than MII-VI oocytes (**Figures 2A, B**). Intracytoplasmic ROS levels were similar among the three groups (**Supplementary Figures 1A, B**). Elevated mitochondrial ROS promotes early apoptosis in oocytes (20). We observed a significantly higher proportion of oocytes undergoing early apoptosis compared to CON and MII-VI groups (**Figures 2C, D**). Oocytes in the GV-VI group exhibited a higher spindle length-to-width ratio compared to the CON and MII-VI groups (**Figures 2E, F**). These findings suggested that vitrification at the GV stage resulted in significant mitochondrial ROS accumulation, increased apoptosis incidence, and abnormal spindle elongation, which might be associated with functional impairment.

### 3.3 Vitrification at GV stage compromises the mitochondrial function

**Figure 3:**
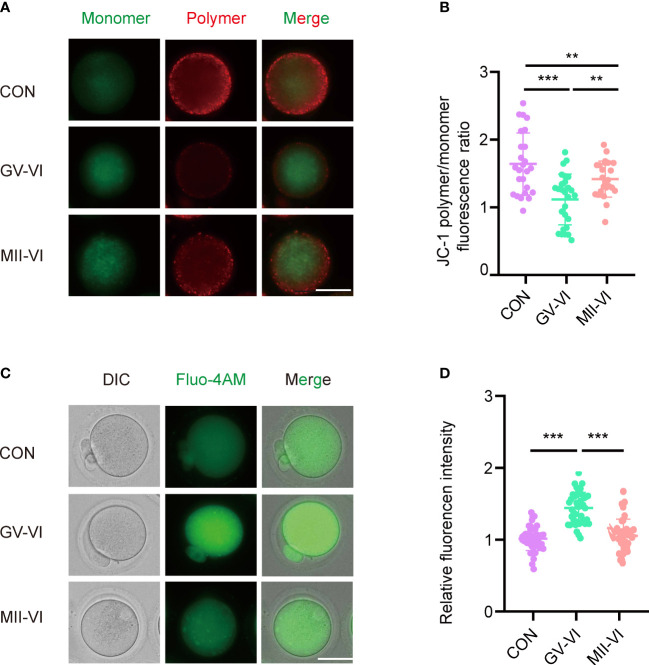
Effects of vitrification at different meiotic stages on mitochondrial function. **(A)** Mitochondrial membrane potential (ΔΨm) was detected through JC-1 staining in oocytes in the CON, GV-VI, and MII-VI groups (red, polymer; green, monomer). Scale bar, 25 μm. **(B)** The ratio of JC-1 polymer (red) to monomer (green) fluorescence was in oocytes in the CON (n = 25), GV-VI (n = 27), and MII-VI (n = 24) groups. **(C)** Representative images of intracellular calcium levels were detected using Fluo-4 AM staining. Scale bar, 25 μm. **(D)** Fluorescence intensity of intracellular calcium ions level measured in oocytes in the CON (n = 39), GV-VI (n = 40), and MII-VI (n = 37) groups. Data in **(B)** and **(D)** are presented as the mean ± SEM, from at least three experiments, **p < 0.01, ***p < 0.001.

Mitochondria play a critical role in cellular metabolism and energy production, with dysfunction linked to ROS accumulation and cell apoptosis (21). Considering the immediate damage caused by vitrification and warming, we assessed the distribution of mitochondria of oocytes from the MII-VI group. The results showed that the distribution of aggregated mitochondria caused by vitrification and warming was repaired by oocyte incubation for 2.5 hours following the warming (**Supplementary Figures 2A, B**). Then we assessed the mitochondrial membrane potential of MII-stage oocytes, observing a significant decline in the GV-VI group compared to the CON and MII-VI groups (**Figures 3A, B**). Given that mitochondria regulate calcium homeostasis (20), we measured intracellular calcium levels and found higher levels in the GV-VI group compared to the CON and MII-VI groups (**Figures 3C, D**). Based on these findings, we concluded that vitrification at the GV stage impairs mitochondrial function.

### 3.4 Vitrification at GV stage influences maternal mRNA degradation during oocyte maturation

**Figure 4:**
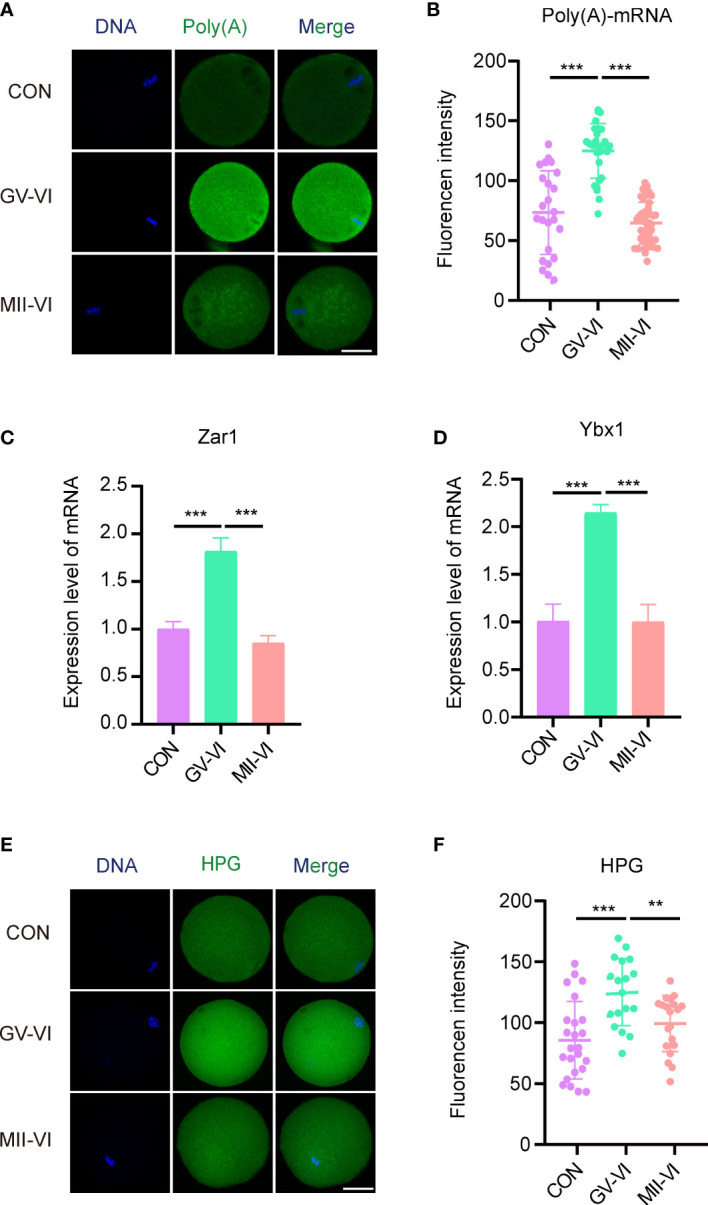
Effects of vitrification at different meiotic stages on the degradation of maternal mRNA during IVM. **(A)** Representative images of the cytoplasmic distribution and abundance of poly(A) mRNA, assessed using a 5′-FITC-oligonucleotide(dT)20 probe, of oocytes in the CON, GV-VI, and MII-VI groups. Scale bar, 25 μm. **(B)** Fluorescence intensity of poly(A)-mRNA in oocytes from the CON (n = 25), GV-VI (n = 27), and MII-VI (n = 32) groups. **(C, D)** Expression levels of Zar1 and Ybx1, determined by RT-PCR in the CON, GV-VI, and MII-VI groups. **(E)** Representative images of protein synthesis, detected by HPG staining, in oocytes from the CON, GV-VI, and MII-VI groups. Scale bar, 25 μm. **(F)** HPG fluorescence intensity in oocytes from the CON (n = 24), GV-VI (n = 21), and MII-VI (n = 21) groups. The data in **(B–D, F)** are the mean ± SEM, from at least three experiments. **p < 0.01, ***p < 0.001.

During oocyte maturation, mRNA levels significantly decrease, and abnormal degradation is associated with early developmental blocks (22, 23). We measured poly(A) mRNA abundance and found that MII oocytes in the GV-VI group had significantly higher contents compared to the CON and MII-VI groups (**Figures 4A, B**), indicating defective maternal mRNA degradation. We further verified the delay of maternal mRNA decay in the GV-VI group by assessing the expression of two maternal genes, Zar1 and Ybx1, which are crucial regulators of maternal-to-zygotic transition (24, 25) (**Figures 4C, D**). Protein synthesis in MII oocytes was also assessed, revealing significantly higher L-HPG levels in the GV-VI group compared to the CON and MII-VI groups, confirming defective maternal mRNA degradation after vitrification at the GV stage (**Figures 4E, F**). Our results thus demonstrate that vitrification at the GV stage hinders maternal mRNA degradation during oocyte maturation, potentially leading to oocytes with a decline in developmental competence.

### 3.5 Vitrification at GV stage reduced developmental competence

**Figure 5:**
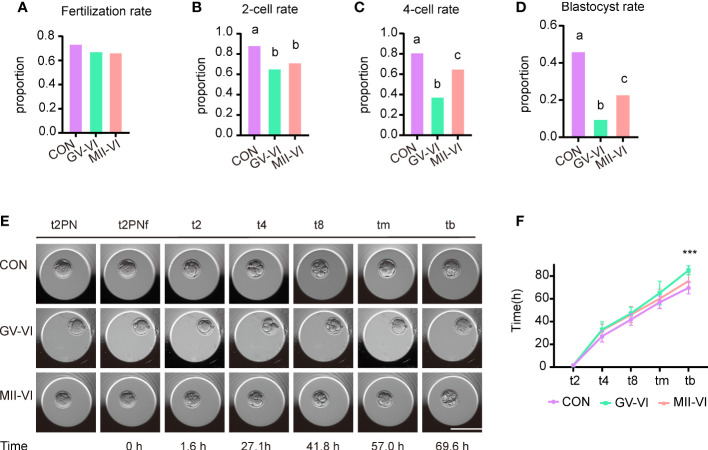
Effect of vitrification at different meiotic stages on embryo developmental competence. **(A)** Fertilization rates (proportion of 2PN relative to total oocytes) in the CON (n = 140), GV-VI (n = 136), and MIII-VI (n = 164) groups after ICSI. **(B)** The 2-cell rates (proportion of 2-cell to total 2PN embryos). **(C, D)** The proportions of the 4-cell and blastocyst stages (proportions of 4- to 2-cell embryos, and blastocysts to 2-cell embryos, respectively). **(E)** Representative time-lapse images from the three groups at the indicated stages. 2PN, two pronuclei; t2PNF, time until the two pronuclei fade; t2, t4, t8, tm, and tb, time (in hours) for the embryo to reach the indicated embryonic stages post-t2PNF. Scale bar, 100 µm. **(F)** The time between pronuclei fading and each stage in the CON (n = 16), GV-VI (n = 6), and MII-VI (n = 12) groups. Values labeled with different letters differ significantly, based on the χ2 test (p < 0.05). Data in **(F)** are presented as mean ± SEM, from at least three experiments. ***p < 0.001.

We subsequently compared fertilization rates and early embryonic development among the three groups. The ICSI experiment demonstrated similar fertilization rates across the groups (CON: 73.6%, GV-VI: 68.4%, MII-VI: 66.5%, p = 0.392; **Figure 5A**). However, the rates of 2-cell (CON: 88.4%, GV-VI: 65.6%, MII-VI: 71.6%; p = 0.001), 4-cell (CON: 81.3%, GV-VI: 37.7%, MII-VI: 65.4%; p < 0.001) and blastocyst (CON: 46.2%, GV-VI: 9.8%, MII-VI: 23.1%, p < 0.001) formation were higher in the MII-VI group than GV-VI group (**Figures 5B–D**). The developmental competence of embryos was lower in both vitrification groups compared to the CON group. Notably, over 60% of embryos in the GV-VI group were arrested at the 2-cell stage (**Figure 5C**). In addition, time-lapse monitoring showed that the embryos in the GV-VI group developed slower than the CON and MII-VI groups (**Figures 5E, F**). Our results suggest that vitrification at the MII stage after IVM may offer enhanced developmental competence compared to vitrification at the GV stage, followed by IVM after warming.

### 3.6 Transcriptomic analysis of differentially expressed genes in 2-cell embryos

**Figure 6:**
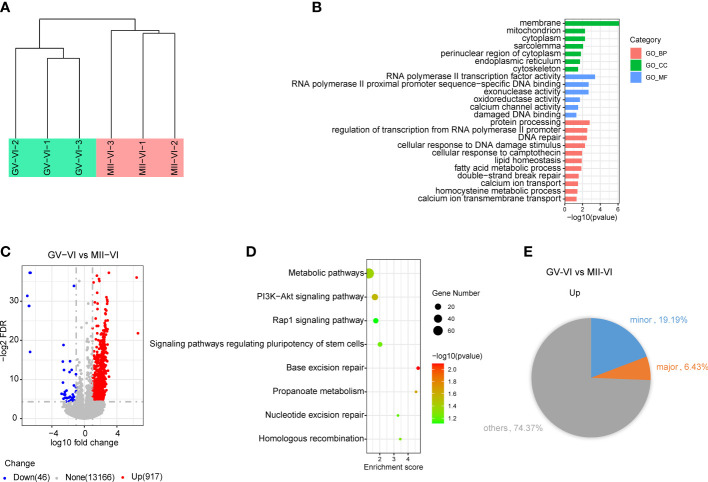
Effect of vitrification at different meiotic stages on the gene expression profiles of 2-cell embryos. **(A)** Unsupervised hierarchical clustering analysis of global gene expression profiles in 2-cell embryos from the GV-VI and MII-VI groups. **(B)** Scatter plot showing changes in transcript levels in 2-cell embryos from the GV-VI and MII-VI groups. Upregulated and downregulated genes in GV-VI are highlighted in red and blue, respectively. **(C)** GO enrichment analysis of cellular components (CC), molecular functions (MF), and biological processes (BP) of DEGs. **(D)** KEGG enrichment analysis of upregulated DEGs. **(E)** Pie chart showing percentages of minor and major ZGA genes upregulated in 2-cell embryos from the GV-VI group.

To determine the mechanism responsible for the reduced developmental competence observed in the GV-VI group, we performed RNA-Seq on 2-cell embryos from both vitrification groups. Unsupervised hierarchical clustering showed strong intragroup consistency and effectively distinguished the GV-VI and MII-VI groups (**Figure 6A**). A total of 963 significant differentially expressed genes (DEGs) were identified (false discovery rate < 0.05, fold change > 10; **Figure 6B**). Among these DEGs, 917 (95.22%) were upregulated in the GV-VI group, suggesting a potential link between abnormal gene induction and reduced in developmental competence.

To further explore the functions of upregulated genes in the GV-VI group, gene Ontology (GO) enrichment analysis was performed. The results of the analysis revealed that the upregulated genes were enriched in mitochondria, calcium channel activity, and DNA damage and repair (**Figure 6C**). Additionally, KEGG pathway analysis identified eight significant pathways, including metabolic pathways, the PI3K-AKT signalling pathway, the Rap1 signalling pathway, base excision repair, and nucleotide excision repair (**Figure 6D**). These findings provided evidence that compromised mitochondrial function and apoptosis of oocytes might be responsible for the observed upregulation in the GV-VI group. Furthermore, genes involved in RNA polymerase II transcription factor activity, RNA polymerase II proximal promoter sequence-specific DNA binding, and exonuclease activity were also upregulated, indicating abnormal transcriptional activity in 2-cell stage embryos from the GV-VI group (**Figure 6C**). It was also found that 19.19% of upregulated genes in the GV-VI group were minor zygotic genome activation (ZGA) genes, suggesting prolonged expression of numerous minor ZGA genes in the late 2-cell stage in GV-VI group embryos (**Figure 6E**). Taken together, our findings suggest that mitochondrial dysfunction, DNA damage accumulation, and excessive minor ZGA gene expression at the 2-cell stage may contribute to the reduced developmental competence and 2-cell-stage arrest observed in the GV-VI group.

## 4 Discussion

The present study aimed to investigate the impact of vitrification at different meiotic stages on mouse oocyte quality and developmental competence. Specifically, GV oocytes were collected from unstimulated mouse ovaries and subjected to analysis. Our findings revealed that oocytes in the GV-VI group exhibited compromised mitochondrial function, defective maternal mRNA degradation, and reduced developmental competence. These results highlight the importance of the meiotic stage in oocyte vitrification and the potential effects on oocyte quality and developmental competence.

Mitochondrial function is crucial for oocyte maturation and developmental competence, as it plays a vital role in ATP synthesis, redox balance, and metabolism (21, 26, 27). The maintenance of mitochondrial membrane potential (ΔΨm) is essential for these functions (28, 29). Our finding revealed that both vitrification groups exhibited significantly reduced mitochondrial ΔΨm, with the GV-VI group showing more severe damage to mitochondrial function than the MII-VI group. We observed that the damage caused by vitrification at the MII stage can be gradually repaired following warming. On the contrary, GV oocytes matured without sufficient recovery time, possibly explaining the decreased mitochondrial function in the GV-VI group. Impaired mitochondrial function can lead to the abnormal accumulation of ROS in the mitochondria, inducing early apoptosis (20, 30, 31). Therefore, the elevated levels of mitochondrial ROS and early apoptosis in the GV-VI group may be associated with mitochondrial dysfunction. Although cytoplasmic ROS is mainly derived from mitochondrial ROS, antioxidants such as glutathione (GSH) within the cytoplasm can eliminate the excessive ROS to maintain cytoplasmic ROS at a low level, which may explain the similar intracytoplasmic ROS levels among the three groups.

Mitochondria also contribute to Ca^2+^ homeostasis by acting as sensors, decoders, and regulators of calcium signalling (32, 33). Elevated levels of intracellular Ca^2+^ have been associated with mitochondrial dysfunction (15). Intracellular calcium homeostasis is maintained by various calcium channels distributed in the plasma membrane, which exhibit meiotic-stage-specific activity (34, 35). Our study found an increased level of intracellular Ca^2+^ only in the oocytes of the GV-VI group. In addition to severe mitochondrial dysfunction in oocytes of the GV-VI group, we hypothesized that GV-stage-specific calcium channels in the plasma membrane might be more vulnerable to vitrification-warming-induced damage than channels active in MII oocytes. During fertilization, sperm entry induces Ca^2+^ oscillations and activates oocytes (36). The significant elevation of Ca^2+^ in the GV-VI group may interfere with this Ca^2+^ oscillation, resulting in insufficient oocyte activation and reduced developmental competence (37, 38).

Besides mitochondrial function, the timely elimination of maternal mRNA during oocyte maturation is also important for oocyte quality and developmental competence (39). After GVBD, polyadenylated mRNAs are rapidly degraded during the maturation process (19). Notably, nearly 70% of maternal mRNA is degraded prior to fertilization, and failure of this degradation results in developmental arrest (40, 41). In our study, an aberrantly elevated level of poly(A) mRNA was observed only in oocytes in the GV-VI group, indicating that vitrification at the GV stage led to insufficient degradation of maternal mRNA during the maturation process.

To explore the mechanisms underlying the decreased developmental competence of the GV-VI group, we performed RNA-Seq on late 2-cell embryos. Interestingly, 95.2% of the DEGs were upregulated in the GV-VI group, suggesting a potential association between reduced developmental competence and the induction of these genes. Enriched terms related to the mitochondrion and ER suggest that the impaired mitochondrial function and abnormal Ca^2+^ signalling in oocytes of the GV-VI group persisted until the 2-cell stage, thereby affecting developmental competence. In addition, the upregulation of genes involved in DNA damage and double-strand break repair might be caused by increased early apoptosis of oocytes. Moreover, the PI3K-Akt signalling pathway identified by KEGG analysis was reported to contribute to regulating apoptosis of cardiomyocytes and other type cells (42–44). We hypothesized this might be an indicator for apoptosis in the late 2-cell embryos, consistent with the oocyte apoptosis in GV-VI group.

ZGA, the initial transcription of the newly formed zygotic genome, occurs between the late 1- and 2-cell stages in mouse embryos, including the minor and major ZGA processes (45, 46). Minor ZGA precedes major ZGA, and proper regulation of the former is crucial for embryo development (47). We found that 19.1% of upregulated genes in the GV-VI group were minor ZGA genes, indicating a prolonged expression of numerous minor ZGA genes in embryos in the GV-VI group. Previous studies have demonstrated that defects in maternal mRNA degradation can lead to abnormal ZGA and reduced embryonic development (23, 48, 49). Thus, we hypothesize that the excessive minor ZGA observed in the GV-VI group may be due to the failure of maternal mRNA degradation. Therefore, we conclude that the observed decline in developmental competence is a consequence of mitochondrial dysfunction, maternal mRNA degradation deficiency, and accumulated apoptosis.

## 5 Conclusion

In conclusion, vitrification at the GV stage leads to severe mitochondrial dysfunction, aberrant maternal mRNA degradation, and abnormal transcriptional activity in 2-cell stage embryos. Consequently, oocyte vitrification at the MII stage following IVM is more advantageous for patients requiring immediate FP compared to vitrification at the GV stage.

## Data availability statement

The original contributions presented in the study are included in the article or **Supplementary Material**. Further inquiries can be directed to the corresponding author. The RNA-seq data represented in the study are deposited in the NCBI public database at this UTR link: https://trace.ncbi.nlm.nih.gov/Traces/?view=study&acc=SRP434522.

## Ethics statement

The animal study was reviewed and approved by Chongqing Health Center for Women and Children (The ethics committee approval number: 2022001).

## Author contributions

JLi and GH conceived the project. DD and JX designed and analyzed experiments. LZ performed the bioinformatics analysis. DD, YT, JX, and XL performed the experiments. DD and JLi contributed to the manuscript drafting with the help of all the authors. All author contributed to the article and approved the submitted version.
