# Supplementary material for: Effects of meiotic stage-specific oocyte vitrification on mouse oocyte quality and developmental competence
Source: Front Endocrinol (Lausanne). 2023 Jun 26;14:1200051. doi: 10.3389/fendo.2023.1200051 (PMC10338221; doi:10.3389/fendo.2023.1200051)
Supplement: Supplementary file 1 [file DataSheet_1.pdf]

Supplementary Material

Effects of meiotic stage-specific oocyte vitrification on mouse oocyte quality and developmental competence

Dongmei Deng[1, 2 *], Juan Xie[1, 2*], Yin Tian[1, 2], Ling Zhu[1, 2], Xuemei Liu[1, 2], Junxia Liu[1,2], Guoning Huang[1, 2], Jingyu Li[1, 2]

*** Correspondence:** Jingyu Li: cqtnljy@gmail.com; Guoning Huang: gnhuang217@sina.com

1 Supplementary Figures

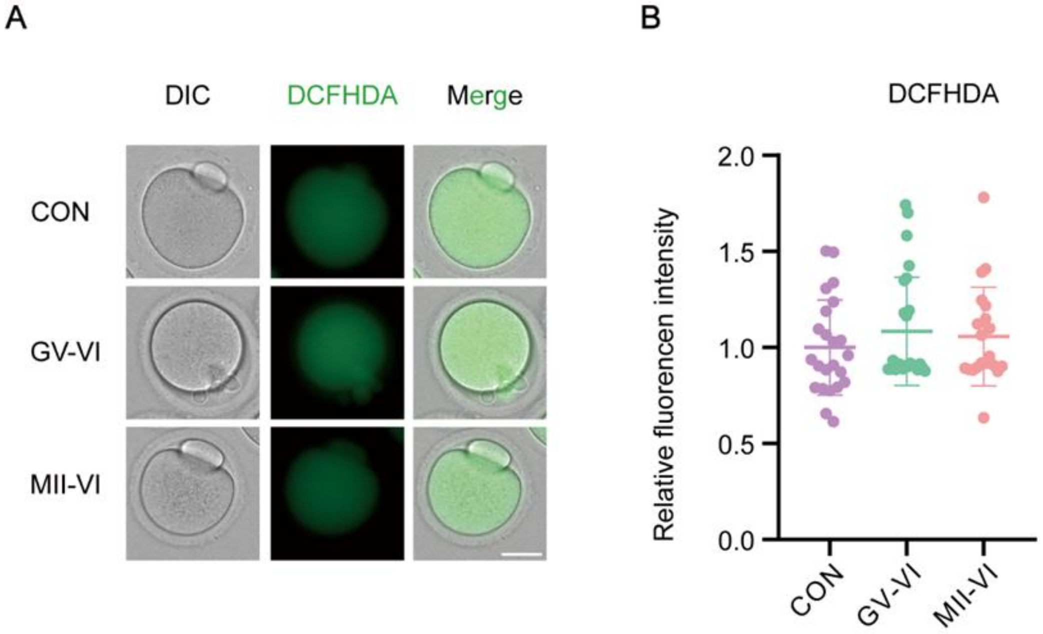

Supplementary Figure 1. Effect of vitrification at different meiotic stages on intracellular ROS. (A) Representative images of intracellular ROS detected through DCFH-DA staining. Scale bar, 25 μm. (B) Fluorescence intensity of ROS signals in oocytes from the CON (n = 24), GV-VI (n = 23), and MII-VI (n = 22) groups. Data in (B) are presented as the mean ± SEM, from at least three experiments, no significant differences were detected by one-way ANOVA with Games-Howell's multiple comparisons test.

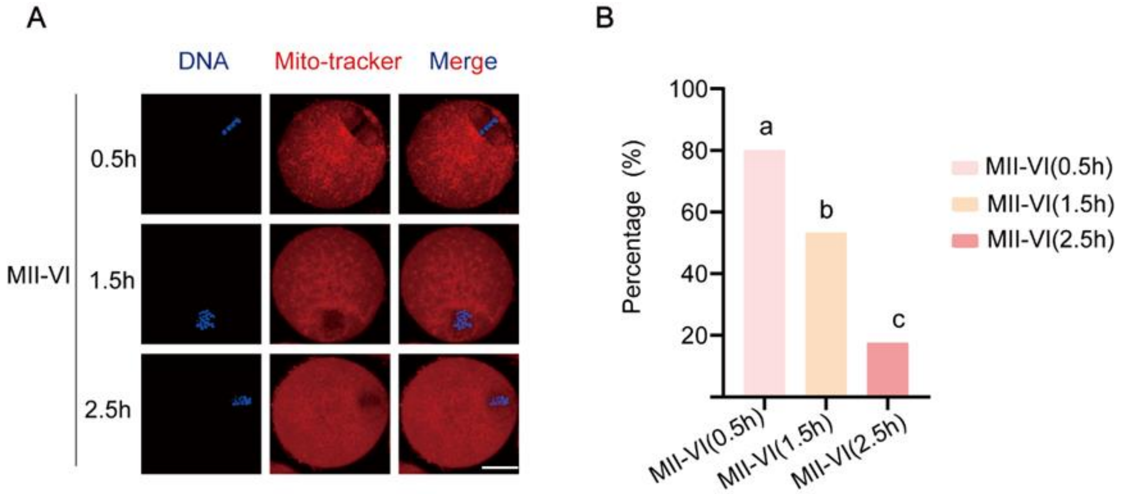

Supplementary Figure 2. Mitochondrial distribution of oocytes from MII-VI group during incubation after thawing. (A) Representative images of mitochondrial distribution were detected through Mito-tracker staining of oocytes in the MII-VI group at 0.5 h, 1.5 h, and 2.5 h after thawing. Scale bar, 25 μm. (B) The percentage of oocytes with abnormal mitochondrial distribution in the MII-VI group at 0.5 h (n = 14), 1.5 h (n = 12), and 2.5 h (n = 15) after thawing. Values labeled with different letters differ significantly, based on the $\chi 2$ test ($p < 0.05$).
